# THE ROLE OF S100B IN AEROBIC TRAINING EFFICACY IN OLDER ADULTS WITH MILD VASCULAR COGNITIVE IMPAIRMENT

**DOI:** 10.1093/geroni/igz038.611

**Published:** 2019-11-08

**Authors:** Rachel A Crockett, Cindy Barha, Ging-Yuek Robin Hsiung, Teresa Liu-Ambrose

**Affiliations:** University of British Columbia, Vancouver, British Columbia, Canada

## Abstract

Aerobic training improves cognitive and brain outcomes across different populations and neurocognitive disorders of aging, including mild subcortical ischemic vascular cognitive impairment (SIVCI). However, little is known of the underlying mechanisms through which aerobic training exerts its beneficial effects on the brain. Recently, S100 calcium-binding protein B (S100B) has been proposed as a possible mediator of aerobic training. At low levels, S100B is neurotrophic but at higher levels it is neurotoxic. Elevated levels of S100B have been associated with decreased performance on measures of global cognitive function. Thus, we conducted a secondary analysis of data collected from the proof-of-concept single-blind randomized controlled trial (NCT01027858) in older adults with mild SIVCI to determine whether the beneficial effects of 6-months, thrice weekly, moderate intensity aerobic training on cognitive performance is related to changes in S100B levels. In a subsample of 45 participants, blood samples were collected both before and after trial completion. Global cognitive function was assessed using Mini Mental State Examination (MMSE). At trial completion, aerobic training decreased circulating levels of S100B compared with usual care plus education (F(1,41) = 6.673, p = 0.013, ηp2 = 0.140; Figure 1). Furthermore, reduced S100B levels were associated with improved global cognitive function in those who received the aerobic exercise intervention (partial r = -0.519, p = 0.023). Together these findings suggest that S100B is a promising target mediating the beneficial effects of moderate-intensity aerobic training on brain health in older adults with mild SIVCI.

